# Assessment of examiner leniency and stringency ('hawk-dove effect') in the MRCP(UK) clinical examination (PACES) using multi-facet Rasch modelling

**DOI:** 10.1186/1472-6920-6-42

**Published:** 2006-08-18

**Authors:** IC McManus, M Thompson, J Mollon

**Affiliations:** 1 Department of Psychology, University College London, Gower Street, London WC1E 6BT, UK; 2 MRCP(UK) Central Office, 11 St Andrews Place, Regents Park, London NW1 4LE, UK

## Abstract

**Background:**

A potential problem of clinical examinations is known as the hawk-dove problem, some examiners being more stringent and requiring a higher performance than other examiners who are more lenient. Although the problem has been known qualitatively for at least a century, we know of no previous statistical estimation of the size of the effect in a large-scale, high-stakes examination. Here we use FACETS to carry out a multi-facet Rasch modelling of the paired judgements made by examiners in the clinical examination (PACES) of MRCP(UK), where identical candidates were assessed in identical situations, allowing calculation of examiner stringency.

**Methods:**

Data were analysed from the first nine diets of PACES, which were taken between June 2001 and March 2004 by 10,145 candidates. Each candidate was assessed by two examiners on each of seven separate tasks. with the candidates assessed by a total of 1,259 examiners, resulting in a total of 142,030 marks. Examiner demographics were described in terms of age, sex, ethnicity, and total number of candidates examined.

**Results:**

FACETS suggested that about 87% of main effect variance was due to candidate differences, 1% due to station differences, and 12% due to differences between examiners in leniency-stringency. Multiple regression suggested that greater examiner stringency was associated with greater examiner experience and being from an ethnic minority. Male and female examiners showed no overall difference in stringency. Examination scores were adjusted for examiner stringency and it was shown that for the present pass mark, the outcome for 95.9% of candidates would be unchanged using adjusted marks, whereas 2.6% of candidates would have passed, even though they had failed on the basis of raw marks, and 1.5% of candidates would have failed, despite passing on the basis of raw marks.

**Conclusion:**

Examiners do differ in their leniency or stringency, and the effect can be estimated using Rasch modelling. The reasons for differences are not clear, but there are some demographic correlates, and the effects appear to be reliable across time. Account can be taken of differences, either by adjusting marks or, perhaps more effectively and more justifiably, by pairing high and low stringency examiners, so that raw marks can be used in the determination of pass and fail.

## Background

An examiner for the MRCP(UK) clinical examination, PACES, in an informal, personal account of examining, wrote:

"Outside, seagulls, starlings, and sparrows, and the occasional blackbird, come and go. Inside, there are hawks and doves." [1]

Clinical examinations require, to a large extent, that judgements of candidates are made by experienced examiners. A potential vulnerability of any clinical examination is that examiners differ in their relative leniency or stringency. Traditionally this is known as the 'hawk-dove' effect, hawks tending to fail most candidates because of having very high standards, whereas doves tend to pass most candidates. Indeed so notorious is the problem that some individual examiners, such as Professor Jack D. Myers ("Black Jack Myers") in the United States, have become famous in their own right as notorious hawks [2]. Although the problem of hawks and doves is easy enough to describe, finding an effective statistical technique for assessing it is far from straightforward.

The hawk-dove nomenclature has itself been criticised (although it must be said that the terms hawk and dove are well-known in the literature, e.g. [3-10]). Alternative suggestions have included 'stringent' and 'lenient', and from a different perspective there is a suggestion that examiners can either be 'candidate centred' (i.e. their sympathies are primarily with the candidates, of whom they wish to pass as many as possible) or 'patient centred' (i.e. their primary aim is to maintain clinical standards at a high level so that patients are protected and provided with competent doctors).

A slightly different approach to naming refers to 'examiner specificity' (e.g. [11]), a candidate's marks depending on the particular examiner(s) they happen to see. The name suggests that this concept is similar to 'case specificity', in which, because candidates are not equally proficient at all clinical tasks they have areas of weakness and strength, and hence can get lucky or unlucky in the particular cases they happen to see, sometimes seeing cases with which they are familiar and other times seeing cases with which, for a host of reasons, they are unfamiliar. Case specificity is said to be found in a wide range of assessment contexts (see e.g. [11-16]), although an important recent study suggests that much case specificity may actually be variance due to items within cases rather than cases *per se *[17]. However, it is not clear that the parallel between case specificity and examiner specificity is in fact appropriate. The key feature of case specificity is that it is a variation in a candidate's ability across different types of case, and so examiner specificity should also refer to a variation in hawkish-dovishness according to the particular case. That though is not what we are referring to here (although it could be analysed), but are instead only considering an examiner's overall propensity for being strict or lenient (in the same way as the overall candidate effect looks at their overall propensity to be correct or incorrect). We will not therefore use the term 'examiner specificity'.

None of the terms is entirely satisfactory, but the hawk-dove nomenclature has the advantage of being in use for at least three decades, and being an effective and easy metaphor (and one which is used in several other areas of science as well, as for instance in games theory and evolutionary biology [18]). Leniency and stringency are however somewhat less emotional descriptors, and we will therefore use the terms leniency and stringency while discussing statistical results, but will also use hawk and dove on occasion when they are useful metaphors in discussion. We must emphasise that when we use the latter terms they should be seen as extremes on a continuum, rather than as discrete classes of individuals (although one does occasionally see comments implying the latter, such as in a surgery examination where is was suggested that "the ratio of hawks to doves is said to be 9:1 or 8:2, so expect at least one examiner of the ten that you meet to appear as 'smiling death' " [19], or in the phrase that, "comparing results across examiners shows that we tend to be either 'hawks' (marking hard) or 'doves' (marking easily)" [20]). However, just as most people are neither extraverts nor introverts, and are neither tall nor short, but instead are somewhere in the middle of the range, so it is likely that most examiners are somewhere between the extremes of being a hawk or dove, and hence are in the mid-range of stringency-leniency. Of course, once stringency-leniency becomes measurable then the shape of the distribution becomes an empirical matter, and will be discussed below.

Although the problem of hawks and doves in medical examination is often mentioned, there are relatively few statistical analyses of the problem (although there is some work within medicine [21,22] and elsewhere [23-25]). An early example of a statistical analysis looking at hawks and doves is to be found in a paper from 1974 which describes a previous major revision of the MRCP(UK) [26]. It considered 10 examinations, taken by 2269 candidates and in whom the overall pass rate was 62.8%. Each candidate was seen by two examiners and together the two examiners produced an agreed mark. "Examiner X" had examined 367 candidates (with 10 different other examiners), and only 46.3% of those candidates had passed the exam, a highly significant difference from the 66.0% pass rate in the remaining candidates (assuming, as the paper says, that candidates were effectively allocated to examiners at random). The paper concludes, "There can be little doubt that X was a 'hawk' whose influence on his colleagues was such as to lower the pass rate for the candidates he examined substantially below the expected level" .

The statistical identification of hawks and doves is not straightforward. At first sight it might seem that examiners could be compared on the average marks they award, with those giving higher marks being classified as doves, and those giving lower marks being classified as hawks. That however assumes that indeed all other things are equal, which is unlikely to be the case. Examiners do not all see the same candidates (and it is possible that candidates in some centres may be less competent than those in other centres). Stations can also differ in difficulty, and examiners not examine an equal numbers of times on each station, so that examining more often on difficult stations might artefactually make an examiner appear to be more hawkish. In this paper we wish to describe a statistical analysis of a large number of candidates who have taken PACES, the clinical examination of the MRCP(UK), in which we use multi-facet Rasch modelling to identify examiner effects.

The examination for the Membership of the Royal Colleges of Physicians of the UK (MRCP(UK)) has always included a clinical examination. In the past the examination took a very traditional format of one long case, several short cases, and an oral examination [27]. In June 2001 the examination was radically restructured into the Practical Assessment of Clinical Examination Skills (PACES) [28]. Before taking the examination, candidates must have passed the Part 1 and Part 2 written examinations, which assess clinical knowledge and applied biomedical science. Selection, training and monitoring of examiners is provided, as described in a document provided by the Colleges [see Additional File 1].

Details of the examination are given in the Method section below, but here it will suffice to say that each candidate receives two separate marks on each of seven different clinical activities. The key to understanding the assessment of examiner stringency in the PACES examination is to realise that each candidate on each station is always seen by two examiners. The two examiners observe the identical clinical encounter at the same time, candidate, centre, patient or simulated patient being seen, clinical task, words spoken by the candidate and the examiners, all being identical. The only thing that differs is the two examiners themselves. If one examiner is more stringent than the other then they will systematically tend to give a lower mark.

If examiners A and B assess together on a number of occasions then a comparison of their paired marks gives an index of their relative stringency. If subsequently B examines with C and then C examines with D, then the paired comparisons allow each of the four examiners to be placed in order, with estimates of the standard errors of their relative stringency. This design is, in effect, an incomplete paired comparison design, and the statistical analysis by the Bradley-Terry-Luce model has been explored for many years [29-31]. In the context of professional sport such models are routinely used for assessing the international ranking of tennis players and chess players based on who has played and beaten whom. The methods are also equivalent to the calculations used in the class of models developed by Georg Rasch (1901–1980), now known as Rasch models [32-34], and which are routinely used for assessing the performance of questions and candidates in a wide range of examinations. In general Rash modelling is straightforward because each candidate will answer every examination question, and item and candidate scores can readily be calculated. That feature is not however necessarily present for assessing examiner effects.

A potential problem for applying Rasch models to examiner stringency is the concept of 'linkage' or 'relatedness'. In a particular diet of an exam, examiners A, B, C and D may have examined together as described above, because they were all working together in a particular centre. At another centre, examiners E, F, G and H may also be working together, and hence an estimate of their relative stringency can also be calculated. However geographical separation, coupled with the practicalities of a single examination at a single point in time, means that none of A, B, C and D ever examines with any of E, F, G and H. The two sets of results from the different centres are therefore not linked, and no estimates can be calculated of the relative stringencies of the entire set of examiners.

A solution to the problem of linkage is found if some examiners examine on several different diets at different centres. If on the next diet, E travels to the other centre and examines with examiner A, then the minimal condition is met for all eight examiners being linked, and a joint analysis of the two diets can rate the stringency of all examiners. The analysis described here considers the first nine diets of the PACES examination, and it will be shown that sufficient examiners have examined with enough other examiners for there to be linkage.

The statistical analysis described here uses the program *FACETS *[35] which carries out Rasch modelling for constructing linear measures from qualitatively ordered counts in multi-facet data. To summarise succinctly, the relationship between a conventional Rasch model (which is now commonly used to analyse the results of examinations) and FACETS, is similar to that of the relationship between simple regression and multiple regression. In simple regression one asks how an outcome variable, such as blood pressure, is related to a background (or independent) measure such as age, whereas multiple regression allows one to see how an outcome measure relates to several background variables, such as age, height, serum cholesterol, and so on. Similarly, while a Rasch model shows how the probability of answering an item correctly on an examination relates to the difficulty of an item and the ability of a candidate, with FACETS one can assess how the probability of answering an item not only relates to item difficulty and candidate ability, but also to a range of background factors, including characteristics of examiners and the nature of the assessment. FACETS, most simply, is therefore a multivariate generalisation of Rasch modelling. That can be seen more clearly in a formal mathematical model.

### The Rasch model

The basic Rasch model considers only a group of *n *candidates, who each have an ability, *C*_*i*_, *(i = 1,n)*, and who each takes a set of *m *tests, each of which has a difficulty *T*_*j*_*(j = 1,m)*. The probability of candidate *i *correctly answering test *j*, *Pij*, is then estimated as:

*logit (P*_*ij*_*) = log(Pij/(1-P*_*ij*_*)) = C*_*i *_- *T*_*j *_    .... (1)

Given a reasonable number of candidates taking a reasonable number of tests it is then possible to use maximum likelihood methods to calculate separately an ability measure for each candidate and a difficulty measure for each test item. In addition, a standard error can be calculated for each of these measures. A practical point to note in equation 1 is that it has used the conventional method of scoring in which higher candidate scores indicate higher ability (and hence a greater likelihood of answering the question being answered correctly), and difficult tests also have a higher score (and hence, because of the negative coefficient in equation 1, a lower probability of being answered correctly). Later this can be seen more clearly in the "yardstick" output from *FACETS*, where the various scores are placed side by side. A very competent candidate climbs high up the diagram, and therefore is successfully answering more difficult stations, and is also satisfying the more hawkish examiners. Rather like a high-jump exam, the better jumpers have a higher chance of clearing the higher jumps.

### The partial credit model

The basic Rasch model considers only items which are answered correctly or incorrectly. However on many forms of examination the examiners rate candidates on a ranking scale (e.g. as in PACES, 'Clear Fail', 'Fail', 'Pass' and 'Clear Pass'). Although conventionally scored as 1,2,3 and 4, there is no statistical basis for treating such judgements as being on an equal interval scale. Such ratings are readily incorporated into the Rasch model, and the size of the intervals can be assessed directly. Let candidates be assessed on a scale with *r *categories, so that each mark has its own difficulty, *M*_*k *_*, (k = 1,r) *. The partial credit model is then:

*logit (P*_*ijk*_*) = log(Pijk/(1-P*_*ijk*_*)) = C*_*i *_- *T*_*j *_- *M*_*k *_    .... (2)

where *P*_*ijk *_is the probability of candidate *i *on test *j *receiving a mark of *k*. Once again the negative coefficient for *M*_*k *_means that high scores for *M*_*k *_mean it is more difficult for a candidate to get a higher mark. The partial credit model allows the differences between the various points on a mark scale to be assessed. (Note, although we here refer to this model as the partial credit model, it is in essence identical to the rating-scale model [36,37]).

### The multi-facet Rasch model

A further extension of the Rasch model allows additional parameters to be estimated which take into account other factors in the design of the test and might account for variability. Although in principle there is no limit to such additional *FACETS*, here we will only consider the situation relevant to PACES, in which examiners also differ in their stringencies. Let there be *p *examiners, each of whom has a stringency, *E*_*l *_(*l = 1,p*), with a high stringency meaning that a candidate is less likely to receive a higher mark from that examiner than they are from a less stringent examiner. The equation then can be expressed as:

*logit (P*_*ijkl*_*) = log(Pijkl/(1-P*_*ijkl*_*)) = C*_*i *_- *T*_*j *_- *M*_*k *_- *E*_*l *_    .... (3)

The probability of a candidate receiving a particular mark then depends on their own ability (*C*_*i*_), the difficulty of the test (*T*_*j*_), how high the mark is (*M*_*k*_), and the stringency of the examiner (*E*_*l*_). In this paper we will restrict ourselves to the model shown in equation 3. Although in theory it is straightforward to include in the model other *FACETS *which might affect the performance of candidates, that is not always easy in practice because the data in complex designs are not always 'linked' or 'connected', where connected is used in the technical sense used in graph theory, in that there is a path between all possible pairs of vertices. For further discussion of this see the *FACETS *manual [35], which also refers to the work of Engelhardt [38], and says that the algorithm for testing connectedness is an extension of that described by Weeks and Williams [39].

The primary interest of the present paper will be in the differences which occur between examiners, and in how these may be estimated, and in ways in which they may be corrected for in the marking of the examination. Examiner variation can reduce the validity of an examination since the likelihood of a candidate passing depends not only upon the candidate's own ability, but also upon whether they got lucky or unlucky in their particular choice of examiners. Although examiners are to a first approximation randomly allocated to candidates, there can also be systematic biasses (and in the case of the PACES examination it is known that candidates sitting in centres outside the UK often have a lower overall pass rate than those taking the examination in UK centres, and it is also the case that the most experienced UK examiners are also the ones who collaborate with local examiners in examining at centres outside the UK). We will also try and assess what demographic factors characterise stringent or lenient examiners, and whether examiners vary in their stringency in different tests of the examination, or with particular types of candidate.

## Methods

The results of the first nine diets of PACES (2001 to 2004) were analysed. In addition and where possible, data were collected on the demography of examiners and candidates, in order to assess their contribution to variation in examiner stringency.

### The PACES examination

**Figure 1:**
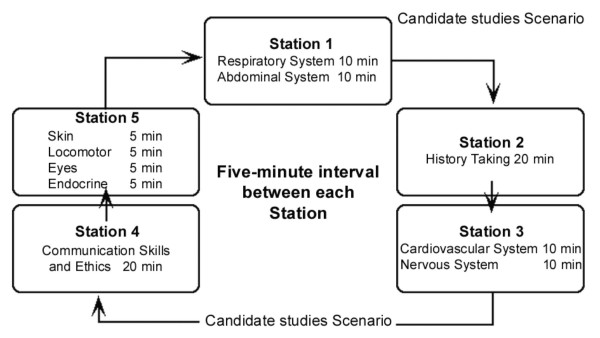
The structure of the PACES examination carousel. Note that candidates can start at any position in the carousel.

The PACES examination for an individual candidate consists of a 'carousel' of five separate stations, each lasting twenty minutes (see figure 1). Two of the stations assess aspects of communications (2: 'History taking' and 4: 'Communication Skills and Ethics') and last 20 minutes. Two of the other stations are each split into two sub-stations lasting ten minutes, which are assessed by the same pair of examiners, and for which candidates receive a separate mark on each part (1: 'Respiratory' and 'Abdominal'; 3: 'Cardiovascular' and 'Neurological'). Finally, station 5 lasts 20 minutes, and consists of Skin, Locomotor, Eyes and Endocrine ('Other'), and there is a single mark for the whole station. Candidates therefore receive a total of fourteen separate marks from ten different examiners (two at each of the five stations). As in an OSCE examination, a candidate may start at any point on the carousel. An important difference from a traditional OSCE is that within each station there is a selection of patients and candidates will differ in which particular patients they happen to see. Examiners are however aware of this, and are specifically instructed before the examination begins to assess the difficulty of the particular symptoms and signs in the patients, and to adjust their marks in relation to that difficulty. For further details, as well as examples of the mark sheets used by examiners, are available on the internet [40].

At each station the candidate is assessed by two examiners, each of whom marks entirely independently of the other examiner, and there is no conferring or discussion after the candidate has left the room. Marking takes place on a proforma on which examiners indicate the candidate's proficiency on a number of sub-scales, and then they make an overall judgement of the candidate. The overall judgement is implicitly criterion-referenced, and has four categories (Clear Pass, Pass, Fail, Clear Fail), with anchor statements describing the performance at each level. It is intentional that there is no judgement between the marks of Pass and Fail, so that examiners explicitly have to make a decision about each candidate, relative to the standards expected of a just-passing candidate taking the examination.

The four categories of Clear Pass, Pass, Fail, Clear Fail receive numerical marks of 4,3,2 and 1. Since each candidate receives a total of fourteen marks, the total mark is between 14 and 56. For various historical reasons, and after development and piloting, the pass mark at the first diet of PACES was set at 41, and has been maintained at that level for the first nine diets, which are reported here. An additional rule, which applies to only a tiny percentage of candidates, is that any candidate who receives three Clear Fail marks from three different examiners will automatically fail the examination; in practice most candidates meeting this criterion would have failed the examination anyway as their total mark is below 41.

Statistical analysis used *FACETS *3.50.0 [35] for Rasch modelling, *Xcalibre *1.10b [41] for fitting two-parameter item-response theory models, and *SPSS *11.5 for all other statistical calculations.

### Meaning of 'stations'

It should be noted that although there are five physically separate stations in the examination proper, for the remainder of this paper the term 'station' will be used rather more conveniently to refer to each of the seven separate assessments made of a candidate, rather than to the five twenty-minute sessions within which those seven assessments are made.

## Results

### Candidate data

The first nine diets of the PACES examination were taken between June 2001 and March 2004, with two diets in 2001, three in 2002 and 2003, and one in 2004. The total number of candidates taking the examination was 10,145, an average of 1,127 on each diet (range 784–1,355). Some candidates took the exam on more than one occasion, and for the present analysis they have been treated as if at each time they were separate candidates (since it was to be expected that their performance may have improved across diets) . On each diet the pass mark was set at 41 (for explanation see Dacre *et al *[42]). Overall the pass rate was 46.6% (4724/10145). Overall the analysis considered a total of 142030 marks.

6834 candidates were male (67.4%) and 3311 were female (32.6%). 4483 (44.2%) of the candidates were graduates of UK medical schools, and 8916 (87.9%) of the candidates were taking the examination in centres based in the UK.

### Examiners

Overall 1259 examiners took part in the 9 diets of PACES, with each examiner being paired in every case with a second examiner. Each examiner had assessed an average of 113 candidates (SD: 83, range 1–593; quartiles = 50–153; median = 96; mode = 40). 1037 examiners (82.4%) only examined candidates in the UK, 119 examiners (9.5%) only examined candidates outside the UK, and 103 examiners (8.2%)examined candidates in both the UK and elsewhere. The latter group had more overall experience of examining in PACES (mean candidates examined = 238; SD = 104), than those examining only in the UK (mean candidates examined = 105; SD = 73), or those examining only outside the UK (mean candidates examined = 73; SD = 44). The average year of birth of examiners was 1951.4 (SD = 6.6, range = 1931 – 1968). 1042 examiners were known to be male, 123 were known to be female, and the database did not have information on the other 94 examiners (60 of whom examined only outside the UK and 34 of whom examined only in the UK).

#### Multi-facet Rasch modelling

##### Separating the effects of candidate ability, test (station) difficulty, examiner stringency and the marking scale

A three-facet Rasch model was run on all 142,030 examination marks, using the model described in equation 3 above. Of particular importance was that *FACETS *reported that subset connection was "OK", meaning that the data were connected and that linkage had occurred satisfactorily, so that examiner stringency could be compared on a common scale across all examiners.

Since subset connection is so important to *FACETS*, we investigated the extent to which it was achieved by smaller data sets. We tried three different sets of just three diets (1,2 and 3; 7, 8 and 9; 1, 4 and 9) and in each case connection was OK. When however we tried just two diets (8 and 9; 1 and 9) we found that while the latter was connected, the former showed 19 disjoint subsets. It seems likely that three diets is necessary for connection to be adequate. We also attempted to run a model using data from just one station, but that analysis failed with multiple disjoint subsets, showing that although case connection is satisfactory for the entire data set, it is vulnerable as soon as only a part of the data is used.

**Figure 2:**
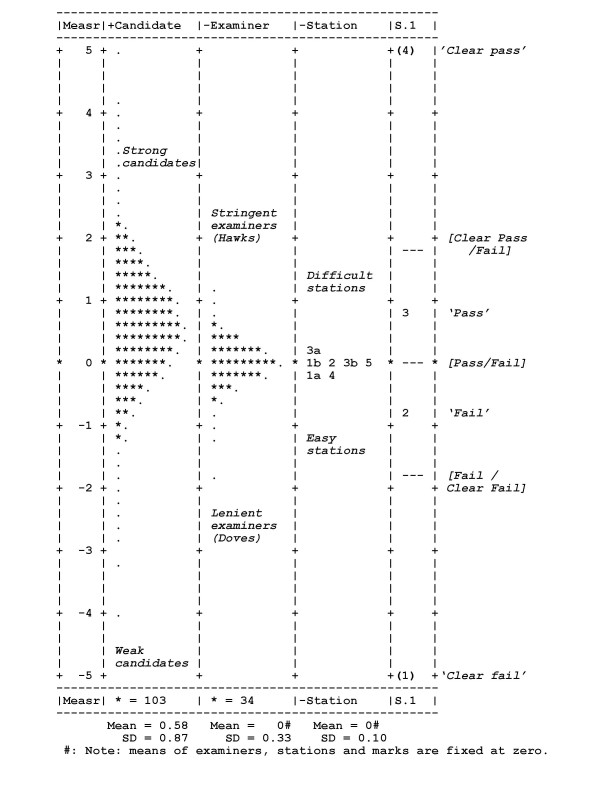
The 'yardstick' comparing marks on a common logit scale for candidate ability, examiner stringency, station difficulty and scaled marks.

Figure 2 shows FACETS' 'yardstick' display of the logit scores for candidates, examiners, stations and marks. It should be emphasised that because all measures are estimated through equation 3, then they are all on a common scale and therefore can be directly compared. The reader is also reminded that high scores correspond to high scoring candidates, stringent examiners, difficult stations, and high marks awarded. The analysis also ensures that estimates for effects of candidate, examiner, station and mark each takes into account any effects due to confounding with other variables. In particular the effects of examiners take into account whether or not those examiners had examined particularly weak or strong candidates, or examined on difficult or easy stations. Effects shown are therefore true effects and are not confounded. Likewise the effects for candidates have been disconfounded for differences in examiners (see below for further discussion).

##### Scale differences

**Figure 3:**
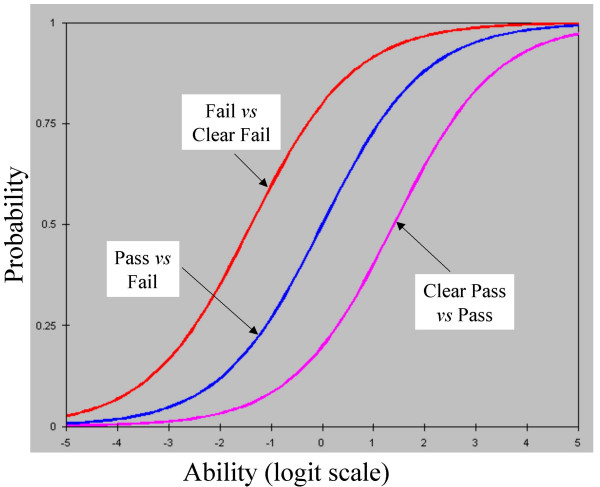
Cumulative probability of a candidate of particular ability crossing each of the scale boundaries between Clear Fail and Fail, Fail and Pass, and Pass and Clear Pass.

**Figure 4:**
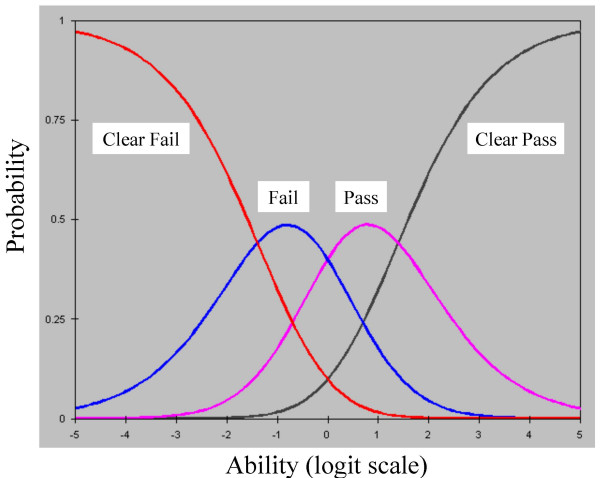
The probability of a candidate of a particular ability achieving each of the four marks on a station of average difficulty with an average examiner.

Taking all of the examiners' marks together, 8% were 'Clear fail', 27% were 'Fail', 40% were 'Pass' and 25% were 'Clear Pass'. The rightmost column of figure 2 shows the positions of the boundaries between these marks on the common logit scale of the Rasch model, at -1.39, 0.00, and 1.30; to a good approximation therefore the scale of marks is indeed equal interval. The numbers '2' and '3' in the rightmost column of figure 2 indicate the modal points of 'Fail' and 'Pass', at -0.79 logits and 0.80 logits. Figure 3 shows the conditional probabilities for the three steps between the four marks, and figure 4 shows the probabilities of the four marks being awarded for candidates at different points along the ability scale.

##### Station (test) differences

Previous analyses of the PACES data have suggested that the stations differ to some extent in their difficulty [42,43], candidates receiving the highest average mark on the Communication and Ethics station, and the lowest average mark on the Cardiovascular station. Table 1 provides the *FACETS *analysis of the station differences, ranked in order from most difficult to least difficult. The first two columns of data show the average mark on each of the stations, and the point-biserial correlation between the mark on each station and on the exam as a whole. The column marked 'Station effect' shows the logit score for each station, and in the next column is shown the standard error of the logit score. The standard errors are all of the order of 0.1. Station 3a (Cardiovascular) is the most difficult, followed by 3b (Neurological) and 1b (Abdominal), which are of similar difficulty. Stations 2 (History taking) and 5 (Other) are easier but of similar difficulty, and stations 4 (Comunication Skills and Ethics) and 1a (Respiratory) are the easiest and of equivalent difficulty. The final column of table 1 shows the average mark on each station adjusted for differences in candidate and examiner mix. These adjusted marks are, however, very similar in pattern to the raw marks. *FACETS *provides an estimate of the reliability of the differences between the logit scores by comparing the standard deviation of the station logit scores (0.10) with the root mean standard errors for those scores (0.1). The reliability for station differences is calculated by *FACETS *as 0.99 (i.e. the differences are highly reliable).

**Table 1:** Differences between the seven stations in average mark, and effect estimated by *FACETS*. The final column shows the average adjusted mark, after taking examiner and candidate differences into account.

	*Station*	*Average (Raw)*	*Point-Biserial*	*Station (test) effect*	*SE of effect*	*Average (adjusted)*
3a	Cardiovascular	2.72	0.30	0.18	0.01	2.76
3b	Neurological	2.78	0.34	0.07	0.01	2.83
1b	Abdominal	2.78	0.30	0.06	0.01	2.83
2	History taking	2.84	0.33	-0.05	0.01	2.9
5	Other	2.84	0.31	-0.05	0.01	2.9
4	Communication and Ethics	2.88	0.34	-0.11	0.01	2.93
1a	Respiratory	2.88	0.33	-0.11	0.01	2.93

##### Examiner differences

The yardstick in Figure 2 shows that examiners are more variable than are stations, the standard deviation being 0.33 for examiners but only 0.10 for stations. However it should also be noted that the SD for candidates is 0.87, meaning that the spread of candidates is 2.64 times that of examiners (and hence the candidate variance is 6.97 times as large as the examiner variance). On the basis of those figures, 87% of the systematic variance in the marks is due to differences in candidates, 12% due to differences in examiners, and 1% due to differences in station type. In interpreting these results it should be noted that the FACETS analysis cannot take into examiner-by-station and examiner-by-candidate variance, and hence any calculation of reliability or similar figures is likely to be inflated relative to the true value. However, these statistics are not the main interest of the present paper, so that the problem is not a serious one.

The yardstick of figure 2 shows that the extremes of examiner stringency are of a similar size to the difference between the Pass and Fail borderlines. The distribution of examiner stringency estimates in the yardstick also makes clear that, to a good first approximation, stringency is normally distributed, and refutes any simple differentiation of examiners into two separate classes who can be called hawks and doves.

**Figure 5:**
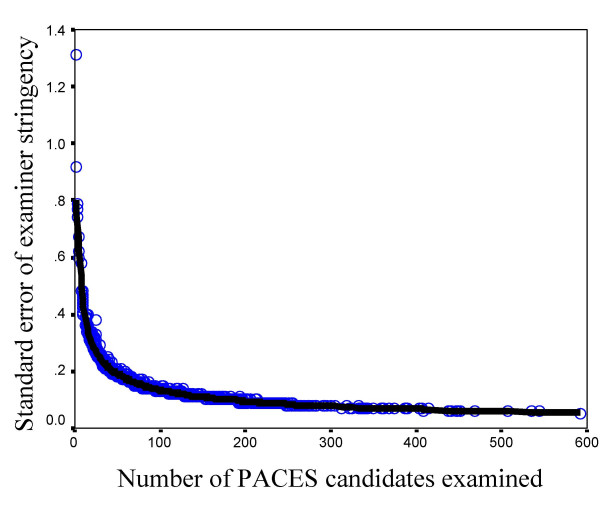
The standard error of examiner stringency/leniency in relation to the total number of candidates examined in PACES.

The accuracy with which examiner stringency is known depends, for obvious reasons, on the number of candidates that the examiner has examined. Figure 5 shows the standard error of the stringency effects in relation to the number of candidates examined. An examiner who has assessed 50 candidates has a standard error of about 0.19, compared with .13 after examining 100 candidates. .09 after examining 200 candidates, and .07 after examining 400 candidates. Stringency measures when only small numbers of candidates have been examined should be treated with care. *FACETS *allows a calculation of the reliability of examiner stringency effects, which it gives as 0.70. That is more than high enough for statistical analysis for comparison of groups, but estimates of the stringency of individual examiners should also be treated with care.

An important question concerns the factors which differentiate between stringent and lenient examiners. Information was available on the sex of examiners and their year of birth, the number of candidates they had examined, and the proportion of candidates who were examined in the UK. These will be considered in turn.

##### Sex

The 1040 male examiners had a slightly higher stringency score (mean = .002; SD = .326) than the 123 female examiners (mean = -.0537; SD = .359), although the difference was not statistically significant (t = 1.774, 1161 df, p = .076).

##### Year of birth

There was no significant correlation of stringency with examiner year of birth (r = -.028, n = 1162, p = .336). Scattergrams showed no evidence of curvilinearity, and neither did quadratic regression show any significant effect.

##### Number of candidates examined

**Figure 6:**
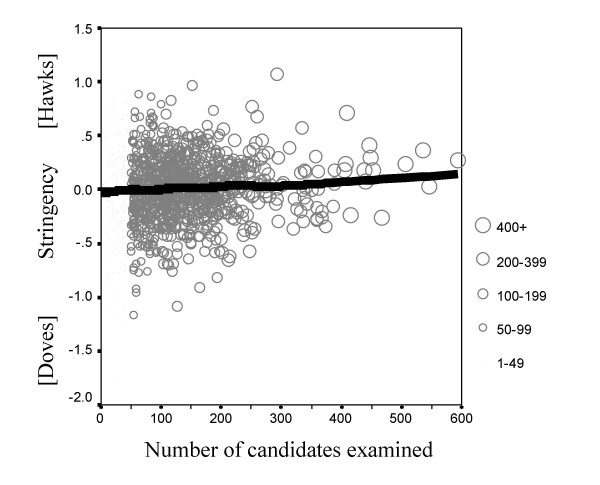
The relationship between examiner stringency (ordinate) and number of candidates examined (abscissa). Examiners who had examined more candidates are shown with larger symbols. The fitted Lowess line shows that stringency increases with the number of candidates examined.

Stringency showed a significant correlation with the number of candidates who had been examined (r = .082, n = 1259, p = .003), which is shown in figure 6. Examiners who had examined more candidates tended to be somewhat more stringent (more hawkish).

##### Proportion of candidates examined in the UK

**Figure 7:**
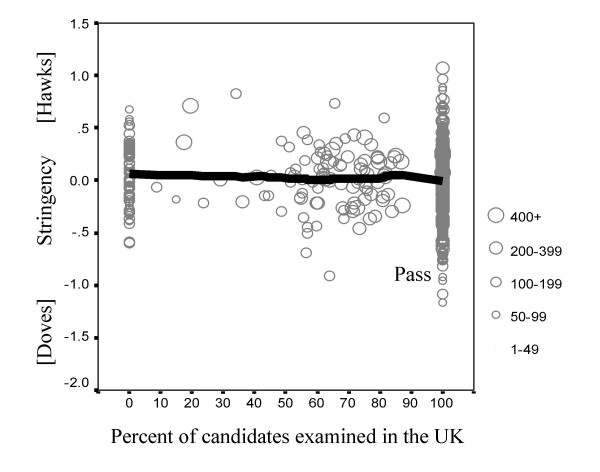
The relationship between examiner stringency (ordinate) and percentage of candidates examined in the UK (abscissa). Examiners who had examined more candidates are shown with larger symbols. The fitted Lowess line shows the relationship between the two measures.

Of the 1259 examiners, 1037 (82.4%) examined only in the UK, 119 (9.5%) examined only outside the UK (in what was usually their home country or a nearby centre), and 103 (8.2%) examined both in and out of the UK, usually being the external examiner sent to a non-UK centre to work with the local examiner. The correlation between stringency and the proportion of candidates examined outside the UK is just significant (r = -.058, n = 1259, p = .039). Figure 7 shows that those examining outside the UK are slightly more hawkish than those examining within the UK, although the effect is small.

##### Ethnic origin of examiners

Self-reported ethnic origin was available for 955 examiners, of whom 84 (8.8%) were non-European and the remainder were European. Ethnic origin was available for only 5 of the examiners who examined entirely outside of the UK, and all 5 were non-European. The 84 non-European examiners had a significantly higher stringency score (mean = .075, SD = .326), compared with the 871 examiners of European ethnic origin (mean = -.0187, SD = .326), the difference being significant (t = 2.509, 953 df, p = .012).

##### Multiple regression

Many of the background factors describing examiners were confounded (e.g. female examiners tended to be younger, and to have examined fewer candidates). The simultaneous effects of sex, year of birth, number of candidates examined, and proportion of candidates examined in the UK were examined by a backwards elimination multiple regression. Missing values were handled by mean substitution. Two effects were independently significant. Examiners who had examined more candidates were more hawkish (beta = .089, p = .005), and examiners of non-European ethnic origin were more hawkish (beta = .079, p = .014). There was no significant sex difference in the multivariate analysis.

##### Candidate differences: reliability of candidate marks

As well as scale, station and examiner differences, the yardstick of figure 2 also shows scores for candidates. It is clear that there is a wide variation in candidate ability, as might be expected. The standard deviation of the candidate logit scores is 0.87 (and the adjusted standard deviation is 0.78), with logit scores in the range of -4.04 to +5.10. The average standard error (root mean square) of the logit scores is 0.37. As a result the reliability of the estimates of candidate ability is 0.82. The standard error on the logit scale for candidates at the pass mark on the raw scale of 41 is about 0.35, which is equivalent to 3 marks on the raw mark scale. That means that an individual candidate scoring 41 has a 95% chance of their true score being two standard errors either side of their actual mark, in the range 35 to 47. As mentioned above, FACETS cannot take into examiner-by-station and examiner-by-candidate variance, and hence estimates of reliability may be inflated relative to the true value.

**Figure 8:**
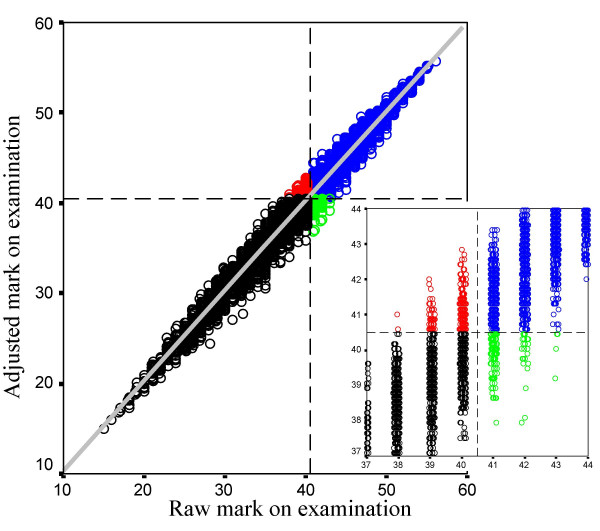
The relationship between the raw mark on the examination and the *FACETS *adjusted mark which takes examiner stringency and station difficulty into account. The pass mark for the examination is indicated by the dashed lines and has been set at 40.5 (see text; i.e. between 40 (fail) and 41 (pass)). Candidates marked in black fail the exam using both raw and adjusted marks, and candidates in blue pass the examination using both raw and adjusted marks. Candidates in green pass the exam on the basis of their raw mark but fail on the basis of the adjusted mark, and candidates in red fail on the basis of their raw mark but pass on the basis of their adjusted mark. The inset figure shows a magnification of the region around the pass mark. It should be noted that a small amount of random horizontal jitter has been applied to the raw marks (which have to be integers) so that the data points are more easily seen.

The candidates' logit scores can be converted back to scores on the same scale as that used by the examiners, and those marks can be adjusted for differences in examiner stringency and station difficulty (which *FACETS *calls 'fair marks' and we will call 'adjusted marks'). Figure 8 shows a scattergram of the adjusted mark for each candidate plotted against the candidate's raw mark. The correlation between the two marks is 0.991. Although the raw and adjusted marks are very similar, they are not always exactly so, some candidates performing better on the adjusted mark, which takes into account the differences in examiner stringency (and these candidates are the ones who saw more stringent examiners).

The conventional pass mark for the examination based on summed raw marks is 41 (although it is probably better described as being 40.5 since candidates with a mark of 41 pass whereas those with a mark of 40 fail, and the true pass mark is somewhere between those two bounds). The vertical and horizontal dashed lines in figure 8 are therefore set at 40.5, and indicate those candidates who would pass or fail using the raw or the adjusted mark. Of the 10,145 candidates, 4568 would pass using either criterion, and 5158 would fail using either criterion. However 263 candidates (2.6%) who failed using the raw mark criterion would have passed using adjusted marks, and 156 candidates (1.5%) who currently have passed the examination would have failed using adjusted marks. The use of adjusted marks would therefore have increased the pass rate from 46.6% to 47.6%, a 1% change in the proportion of candidates passing the examination.

##### Testing the assumptions of the model

Like all statistical models, the multi-facet Rasch model is a simplification of the subtleties of real data. It is useful as a model in so far as a relatively small number of parameters can explain most of the variation present in a complex data. In the present case, 142,030 data points are being explained by a total of 11,412 parameters (10,145 for the 10,145 candidates, 1,258 for the 1,259 examiners, 6 for the seven stations, and 3 for the four scale points), a 92% reduction in information (and equivalent to roughly one fourteenth of the total data).

*FACETS *provides a number of statistics for diagnosing the quality of the fit of the model to the data, of which the manual states that "If mean-squares indicate only small departures from model-conditions, then the data are probably useful for measurement". The manual also says that mean-square statistics in the range 0.5 to 1.5 are desirable, and that those over 2 should be treated with care. There have also been other criticisms of goodness-of-fit statistics derived from Rasch models [44].

##### Examiner goodness of fit statistics

**Figure 9:**
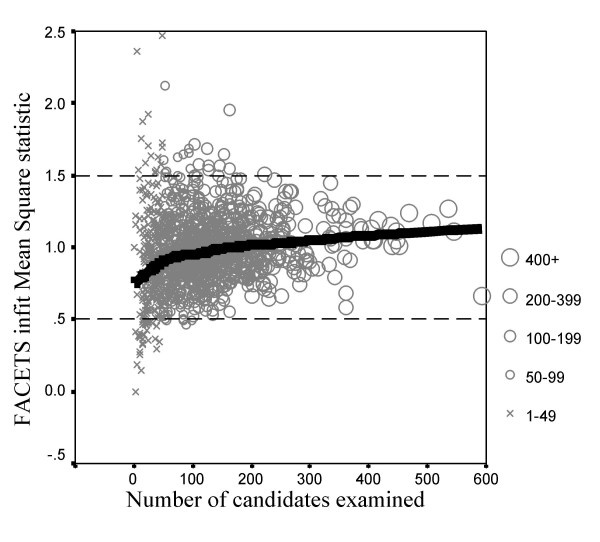
The INFITMS goodness of fit statistics for individual examiners in relation to the number of candidates examined. The solid line shows the fitted Lowess curve. The horizontal dashed lines indicate bounds of 0.5 and 1.5, which indicate acceptable fit.

For the examiner statistics, the INFITMS statistic was larger than 2 in only 3 of the 1259 cases (0.2%), and was larger than 1.5 in only 39 (3.1%) cases. At the other end of the scale, only 38 (3.0%) examiners had values of less than 0.5. Figure 9 shows that the fit of the model improves as the number of candidates examined increases, most of the outliers occurring with those assessing 50 or fewer candidates. Overall therefore the fit of the model to the data from the examiners is good. An important implication is that examiners can rate different stations equally well across candidates (or to put it another way, there is no 'case specificity' at the level of examiners, who are judging all stations equally effectively).

##### Candidate goodness of fit statistics

**Figure 10:**
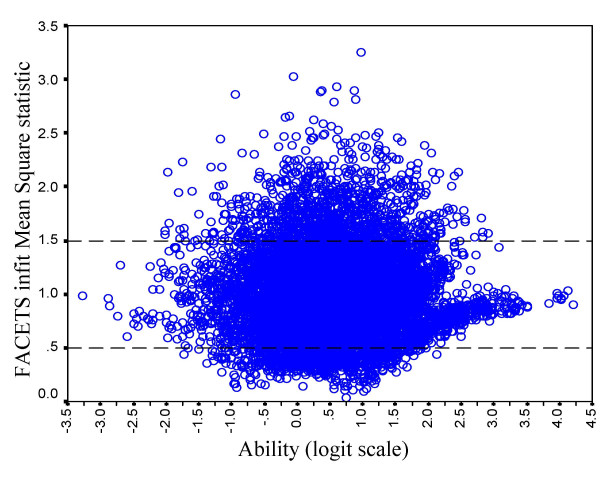
The INFITMS goodness of fit statistics for individual candidates in relation to overall ability. The horizontal dashed lines indicate bounds of 0.5 and 1.5, which indicate acceptable fit.

Figure 10 shows the INFITMS statistic for the fit of individual candidates to the Rasch model in relation to their overall logit score. It is clear that there is a substantial number of cases with values of greater than 2, and that these occur particularly in the middle of the range. This lack of fit probably reflects case-specificity. The Rasch model assumes that candidates differ on a single dimension, and hence ability on each station is determined entirely by overall ability. However a candidate who was particularly good at some stations (perhaps due to specific clinical experience in, say, cardiology or neurology) but was relatively poor at other stations, would not fit the model as well as possible. Such candidates will, of course, be more apparent in the middle of the range since very poor candidates will anyway get low marks on everything, and very good candidates will get high marks on all stations, irrespective of their specific experience.

##### The long-term reliability of examiner stringency measures

**Figure 11:**
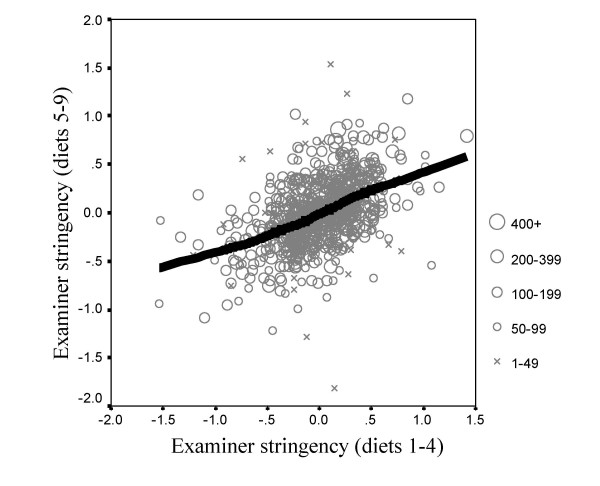
Examiner stringency estimated from diets 1–4 (abscissa) in relation to examiner stringency estimated from diets (5–9). Examiners who had examined more candidates in total across all diets are shown with larger symbols. The solid black line is the fitted lowess curve.

An important question concerns whether the stringency of examiners is a relatively stable feature of their examining behaviour, or whether it varies with time. To answer this question we carried out two separate *FACETS *analyses, one for diets 1–4 (June 2000 – June 2001) and the other for diets 5 – 9 (Oct 2001 – March 2004). In each analysis the program calculated entirely separate estimates of examiner stringency, based on different candidates and different pairings of examiners. Of the 1259 examiners in the overall analysis, 749 had examined both in the earlier period and the later period. Figure 11 shows a scattergram of the stringency estimates for each examiner in the two periods, broken down according to the total number of candidates examined by each examiner in the two periods. The overall correlation is 0.455 (n = 749, p < .001). However this correlation will inevitably be reduced somewhat by the examiners who have examined only a few candidates, and for whom the estimates are less stable. Correlations were therefore calculated separately according to the number of candidates examined. Only 14 examiners had seen 400 or more candidates, and they were therefore merged into the group of examiners who had seen 200 or more candidates. For these 157 examiners, the correlation between the stringency measures in the two periods was 0.598 (p < .001). For the 393 examiners who had seen between 100 and 199 candidates the correlation was 0.533 (p < .001), and for the 152 examiners who had seen 50 to 99 candidates the correlation was 0.411 (p < .001). Finally for the 47 examiners who had seen less than 40 candidates, the correlation was only 0.129 (p = .387). For those examiners seeing large numbers of candidates it is clear that there is an acceptably high correlation of 0.598 between the two independent estimates of stringency. A correlation of 0.598, given that it is based on two estimates based on half as many subjects, is compatible with the overall estimate described earlier of the reliability of examiner stringency measures of 0.70.

We also assessed long-term stability using firstly diets 1–3 and then diets 7–9, since the latter diets were all separated from the former by at least a one-year interval. The within-period reliabilities reported by *FACETS *for these groups of diets were 0.63 and 0.61 (and of course they are lower than those calculated for all nine diets, reported earlier, because they are based on fewer data). The correlations of the stringency estimates across the two periods were 0.402 (n = 146, p < .001) for those examining more than 200 candidates in the entire data, 0.442 (n = 309, p < .01) for those examining 100–199 candidates, 0.335 (n = 101, p < .001) for those examining 50–99 candidates, and 0.468 (n = 20, p = .037) for those examining 49 or fewer candidates overall. These between-period correlations are all compatible with the within-period reliabilities and confirm that stringency is stable across periods of a year or two within the limits of measurement.

##### Differences between 'communication' and 'examination' stations

An important innovation of the PACES examination in the context of the MRCP(UK) was the introduction of two stations which assessed 'communication' rather than 'clinical examination' skills, one assessing history taking and the other assessing how the candidate handled difficult communication and ethical situations. Examiners sometime express concern that they feel less confident in assessing these stations, because of a relative lack of experience in contrast to the 'examination' stations, which assess skills which they all use and assess on a daily basis, and in which they have been proficient for many years. It is therefore of interest to compare the performance of the two communication stations with the five examination stations.

The first question concerns whether the communication stations differ in their difficulty or discrimination as compared with the other stations. The Rasch model used for the multi-facet modelling is a one-parameter Rasch model, and it therefore only allows stations to differ in their overall difficulty (and the analyses reported earlier suggest that there are only relatively small differences in overall difficulty). The Rasch model used by *FACETS *in fact assumes that not only stations, but also examiners, candidates and marks differ only in their difficulty. That assumption cannot be tested directly with *FACETS*, as it does not allow discrimination to differ between the various components of the examination. However two-parameter item response theory (2-IRT) models do allow differences in discrimination between stations [45,46] (although 2-IRT models can only fit a single facet to the data). Here the program *Xcalibre *[41] is used to fit a 2-IRT model to the marks at each station in order to assess the difficulty and the discrimination of each station.

The 2-IRT model fitted by *Xcalibre *has two parameters for each component in the test; the *difficulty*, which is equivalent to the single difficulty parameter fitted in the Rasch model, and the *discrimination*, which allows the slope of the item response curve to differ between stations. A partial credit model was also fitted by fitting three separate binary response measures to each judgement, one for the Clear Fail-Fail borderline, one for the Fail-Pass borderline, and one for the Pass-Clear Pass borderline [47]. For each candidate there were therefore three binary marks derived from each of the fourteen scaled marks, making 42 items altogether. Because each candidate was assessed on each station by two examiners, two marks were analysed for each station, although each was fitted separately. However the two marks which came from each examiner were effectively randomly allocated, the two parameters were expected to be similar, and indeed that was exactly the case. The two parameters from each station at each mark have therefore been averaged for the present analysis.

**Figure 12:**
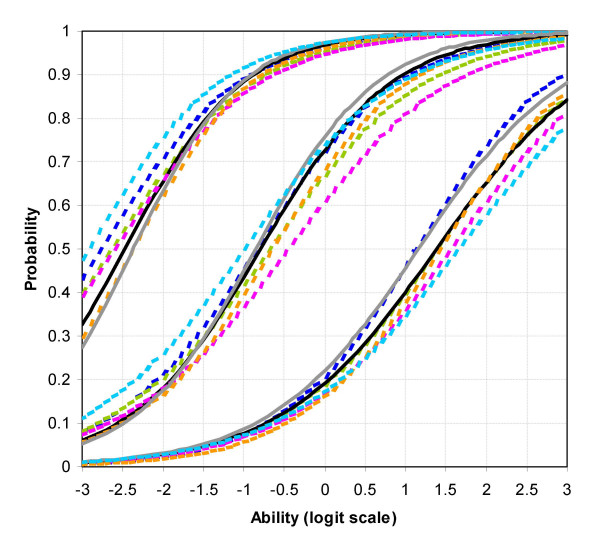
Two-parameter item-response theory curves for the seven stations. The left-most curves are for the Clear Fail – Fail borderline, the middle curves for the Fail-Pass borderline, and the right-most curves for the Pass-Clear Pass borderline. The two communication stations are shown as solid lines (*Black*: Station 2 – History; *Grey*: Station 4 – Communication and ethics), and the five examination stations are shown as dashed lines (*Dark Blue*: Station 1a – Respiratory; *Green*: Station 1b – Abdomen; *Purple*: Station 3a: Cardiovascular; *Orange*: Station 3b: Neurological; *Pale Blue*: Station 5: Other). It should be noted that *Xcalibre *and *FACETS *calculate their parameters in slightly different ways due to making different assumptions to ensure identifiability, and that the output from the programs is therefore not directly comparable (although there is a mathematical relationship between them). This figure should therefore only be used to give a sense of the relative difference between the stations, rather than to be used to compare specific values of the parameters with those derived by *FACETS*.

Table 2 shows the difficulty and the discrimination parameters at each mark level for the seven stations, and figure 12 shows a plot of the item response functions for the marks and the stations. Table 2 shows that there are significant differences between stations in their difficulty and discrimination, since the standard errors are small relative to the differences. However figure 12 shows that in practice the differences have little impact on the overall shape of the curves and that the assumption in Rasch modelling of equal discriminations is unlikely to have invalidated the analysis to any great extent.

**Table 2:** Item difficulty and discrimination parameters fitted by *Xcalibre *to the candidate by station data for PACES. The three separate borderlines for the four points on the mark scale are shown separately. Figures in brackets are standard errors of the estimates. The five examination stations are shown at the top, ranked from most to least difficult on the fail-pass criterion. The two stations at the bottom, in bold, are the communication stations.

	**Difficulty**			**Discrimination**		
Station	Clear Fail-Fail	Fail-Pass	Pass-Clear Pass	Clear Fail-Fail	Fail-Pass	Pass-Clear Pass
3a Cardiovascular	-2.60 (.042)	-0.45 (0.028)	1.56 (0.032)	0.65 (0.021)	0.58 (0.031)	0.60 (0.023)
3b Neurological	-2.37 (.037)	-0.65 (0.025)	1.43 (0.029)	0.80 (0.022)	0.70 (0.027)	0.68 (0.023)
1b Abdomen	-2.64 (.043)	-0.68 (.027)	1.39 (0.031)	0.67 (0.021)	0.61 (0.028)	0.62 (0.024)
1a Respiratory	-2.77 (.045)	-0.85 (.026)	1.13 (0.026)	0.69 (.022)	0.67 (0.026)	0.70 (0.024)
5 Other	-2.92 (.048)	-1.00 (0.028)	1.66 (0.034)	0.73 (0.023)	0.61 (0.026)	0.56 (0.023)
**2 History**	**-2.47 (.039)**	**-0.79 (0.024)**	**1.39 (0.031)**	**0.81 (0.022)**	**0.73 (0.026)**	**0.61 (0.024)**
**4 Communication & Ethics**	**-2.36 (.036)**	**-0.85 (0.023)**	**1.16 (0.028)**	**0.89 (0.023)**	**0.79 (0.025)**	**0.64 (0.023)**

Although a concern of examiners has been that they are uncertain whether they are discriminating well on the communication stations, the fact is that the discrimination parameters at the pass mark are *higher *in the two communication stations (0.73 and 0.79) than in any of the five examination stations (range = 0.58 to 0.70), and that is also the case at the Clear Fail-Fail boundary (Communication stations 0.81 and 0.89; examination stations, range = 0.65 – 0.80). Only at the Pass-Clear Pass boundary are discriminations of the two communication stations (0.61 and 0.64) similar to those in the examination stations (range = 0.56 to 0.70). Overall it can be concluded that examiners are discriminating somewhat *better *on the communication stations, although the differences are relatively small. Certainly there is no evidence that examiners are performing less effectively on the communication stations than on the examination stations.

##### Multi-facet Rasch modelling of communication and examination stations

Since the assumption of equivalent levels of discrimination across stations has been met to a reasonable degree in the different types of station, it is possible to use *FACETS *to examine scores of candidates on just the communication stations or just the examination stations. There are however five examination stations and only two communication stations, making it difficult to have a direct comparison of scores on the two, since inevitably there is less measurement error with five stations. As a result a composite of the two communication stations was compared with a composite of the respiratory and cardiovascular stations. There was somewhat more variation amongst candidates in the communication stations (SD = 1.83) than in the examination stations (SD = 1.43), and as a result the reliability for measurement of candidate ability was slightly higher in the communication stations (.77) than in the examination stations (.69). There was also a little more examiner variation in the communication stations (SD = .78) than in the examination stations (SD = .67), and the examiner reliability was also marginally higher in the communication stations (.70) than in the examination stations (.68). The boundaries between Clear Fail, Fail, Pass and Clear Pass were also slightly further apart in the communication stations (-2.60, -0.05 and 2.65) than in the examination stations (-2.12, 0.10 and 2.02). Taken overall, though, the picture is of remarkable similarity between the communication and the examination stations, with a slightly higher reliability and variability in the communication stations, both amongst candidates and amongst examiners.

## Conclusion

This paper describes an analysis of 10,145 candidates taking the PACES examination over nine diets, when they were examined by a total of 1,259 examiners who awarded a total of 142,030 marks. The multi-facet Rasch model allowed the data to be broken down into three separate components of candidate, examiner and station, along with separate measures for the several components of the four-point marking scheme. Overall the model fitted the data reasonably well, particularly in the examiner facet, although there was some evidence that the model fitted less well for candidates, which may have been due to case-specificity in which candidates differed idiosyncratically, perhaps as a result of different training, on different stations. Nevertheless the latter effects are small, and not relevant to the purpose of the examination as a whole, which requires an overall pass across the different stations.

The multi-facet Rasch model has some limitations, which should be emphasised strongly in considering the current analysis. In particular, FACETS, unlike generalisability theory, cannot consider variance due to interaction effects, primarily because with, say, many candidates and many examiners, there is an extremely large number of degrees of freedom relating to interaction effects (and hence an extremely large number of dummy variables is needed), and it is unrealistic to attempt to estimate so many parameters. Generalisability theory approaches this estimation problem in a very different way, and such interaction effects can be calculated, given certain design constraints (which unfortunately are not applicable here), and the variance terms are often found to be significant and meaningful in examination situations. As a result the estimates provided in this paper of total variance, and the contribution of various facets, may be inaccurate, and should be treated with care. In particular, they probably should not be used for calculating the overall reliability of the examination, or similar statistics. However, and it is an important however, the major interest of this study is in differences between examiners in leniency-stringency, and those differences are primarily likely to be main effects, which FACETS can handle appropriately. There might also be additional variance consisting of interactions between examiners and other aspects of the examination (such as candidates or cases), and future work needs to look for such effects using different methodologies, but the main effects analysed and discussed here are unlikely to disappear in any such analyses. The FACETS analysis is therefore appropriate and adequate as a first approach to studying examiner effects on leniency and stringency.

The principle interest of this study is in differences between examiners. Humans differ in many behavioural attributes, and it is hardly surprising that examiners also differ in their propensity to pass or fail candidates. This study of hawks and doves amongst examiners found highly significant differences in examiner behaviour, which subsidiary analyses showed were consistent across time (within the limits of the reliability of the measures). Examiner variance accounted for about 12% of the systematic variance (as compared to only 1% depending on differences in difficulty of stations, and 87% depending on differences between candidates). Nevertheless these differences are meaningful, particularly to a borderline candidate for whom random allocation of examiners happens to mean that a majority of examiners assessing them could be construed as 'hawks'. *FACETS *allows for raw marks to be adjusted for differences in stringency between examiners. If the PACES examination is re-marked using adjusted marks then about 4% of candidates would change their result across the pass-fail boundary, slightly more going up than down, so that the overall pass rate would increase slightly from 46.6% to 47.6%.

The decision as to whether or not to adjust marks for examiner stringency depends on a number of factors, and the decision is not an easy one. Several factors come into play:

1. The reliability of the adjustments for examiner stringency is easier to apply as more examiners assess more candidates across more diets. It is not technically possible to adjust the results of a single diet of PACES because one cannot obtain linkage across the various subsets of examiners (and even if it were possible, the result would be less reliable than a correction based on as much data as possible on the behaviour of examiners, based on all examinations in which they had taken part). In passing it should be said that it might be possible to obtain linkage within a single diet if linkage could be obtained across stations, perhaps by using a simulator or video station which was objectively marked and therefore of fixed difficulty either for all candidates or for large groups of candidates across examination centres. That is not though possible at present.

2. If examiner stringency can only be assessed reliably across multiple diets then correction for stringency does require that stringency is a stable characteristic of examiners. The comparison of examiners in diets 1–3 with those in diets 7–9 suggests that there is reasonable stability in stringency across a period of a year or two, although that needs to be further examined.

3. It is sometimes suggested that examiners who are 'hawks' or 'doves' should be given feedback about their propensity for marking or high in order that they can then try and correct that tendency. The present analysis would in fact require the precise opposite. It is better given the method of analysis that examiners do not try and correct any differences in stringency, but instead they continue to behave as they have always done. Biasses of any sort which are fixed and unchanging can be corrected statistically, whereas biasses which are varying are, of their very nature, difficult to predict and correction will be less reliable (and hence less valid and less justifiable).

4. There might be an argument for pairing examiners on the basis of their stringency, so that if a candidate sees one examiner known to have a high stringency then the other will have a relatively low stringency. Whether that would be practicable given the complex constraints of a real examination is not clear, but it might be worth investigating. The clear advantage would be that the marking of the examination could then be based on raw scores, which have a high degree of face validity and are easy to justify.

Although there seems little doubt that examiners do differ in their stringency, it is much less clear where those differences come from. Because our sample has a large sample of more than a thousand examiners it is possible to assess the role of several background factors. Important negative results are that we could find no sex differences, and neither did there seem to be any relationship to age, older examiners not being more hawkish than younger examiners. Examiners who had examined more candidates were more hawkish, although whether that is the result of experience making them more hawkish, or more hawkish examiners choosing to examine more often is not clear. Likewise our data suggest that UK examiners from minority ethnic groups are also more hawkish, and again we have no explanation for that, although we did find some evidence for a similar effect in a different analysis [43]. An interesting and important analysis would be to assess how the ethnic origin of an examiner and a candidate interact, but as yet that analysis has not been possible for a host of technical reasons. We are however working on it.

The reasons for differences in examiner stringency could form the basis for a number of future studies. If, as seems possible, stringency is a relatively stable trait then it might be predicted that it would relate to other aspects of personality or behaviour, and in particular the Big Five, which have been shown to relate to many and varied aspects of human behaviour [48,49]. We would hope to address this issue in a future study.

The use of *FACETS *has allowed a full analysis of the marks from nine diets of the PACES examination, it has allowed the separate and independent estimation of effects due to candidate, examiner and station type. As a result it allows a fuller discussion of the origins of examiner effects, and on ways in which the examination might be revised. A point of some importance in the context of designing examinations is that we would not have been able to carry out the present analysis if each station had been assessed by only a single examiner. A recurrent suggestion within the literature on the design of clinical examinations, usually driven by analyses based on generalisability theory, is that when one is trying to maximise the reliability of an OSCE-style examination, "where rater availability is a limiting factor to increasing test length [due to scarcity and expense], more can be gained by using one rater per station and having more stations than using two raters per station" [50]. Although that seems a reasonable strategy, it has two potential problems. Firstly, it does assume that examiner behaviour remains unchanged when only one examiner is present rather than two. However the presence of another examiner, and the potential for cross-checking between independently given marks, may well encourage each of the examiners to be more careful in carrying out their task, and that a lowered examiner reliability for examiners working individually may mean that the overall exam reliability does not increase as much as might be predicted from theoretical calculations. Secondly, and it is one which is particularly relevant to the present analysis, the use of a single examiner at each station does not allow any statistical evaluation of hawk and dove effects, with the likelihood that such effects may well increase in the absence of effective monitoring.

The figurative description of behavioural differences by using animal names is nothing new, the use of hawk to describe, "a person who advocates a hard-line ... policy", goes back to at least 1548, although intriguingly such hawks were contrasted with a range of animals including beetles (1824) and pigeons (1843), whereas the modern contrast with doves only came into use in 1962 at the time of the Cuban Missile Crisis, when, "The hawks favored an air strike to eliminate the Cuban missile bases... The doves opposed the air strikes and favored a blockade." (*Oxford English Dictionary *online [51]). The earliest usages of which we are aware in the context of medical education are both from 1974 [7,26], with one of them concerning the MRCP(UK) examination [26]. However, the problem of hawks and doves amongst examiners is not a new one, and has been described for a century or more in education [52], under a number of different names. Hawks and doves were described as 'the Vulture' and 'the Husbandman', by A C Hilton in a poem written in 1872 [27], and variants of the Hawk were described as 'the Spider' and 'the Poultryman' in a 1904 poem by T C Dent, a surgical examiner [53]. In 1913, Sir William Osler referred to 'Metallics', with their "aggressive, harsh nature and ... hard face", whose, "expression sends a chill to the heart of the candidate, and it reaches his bone marrow [with ] the first question...", to be contrasted with the 'Molluscoid', the "invertebrate examiner, so soft and slushy that he has not the heart to reject the man". Nevertheless, Osler recognised that, "between the metallic and the molluscoid is the large group of sensible examiners" [54]. Despite the long-running awareness of the hawk-dove problem in medical examinations, we are not aware of any previous study which has used a rigorous statistical method to assess properly the stringency or leniency of large numbers of examiners, and to examine how background factors relate to stringency and leniency.

## Conclusion

There is little doubt from these data, that examiners do differ in their leniency or stringency, and the effect can be estimated using Rasch modelling. The reasons for the differences are not so clear, although there are some demographic correlates, and the effects appear to be reliable across time. Various ways are suggested by which account may be taken of differences, either by adjusting marks or, perhaps more effectively and more justifiably, by pairing high and low stringency examiners, so that raw marks can then be used in the determination of pass and fail. The performance of the PACES examination is under continual review by the Colleges, and the implications of these and other findings for the running of the examination are a part of that review

## Abbreviations

FACETS: This is not an abbreviation or an acronym but the name of a computer program

MRCP(UK): Membership of the Royal Colleges of Physicians of the United Kingdom

OSCE: Objective Structured Clinical Examination

PACES: Practical Assessment of Clinical Examination Skills

SD: Standard deviation

SE: Standard error

UK: United Kingdom of Great Britain and Northern Ireland

## Competing interests

ICM is Educational Advisor to the MRCP(UK), and has been an examiner for the MRCP(UK) Part 1 and Part 2 (but not PACES). MT and JM are employees of the MRCP(UK).

## Authors' contributions

JM and MT were primarily responsible for the collection and collation of data; ICM was responsible for the statistical analysis and wrote the first draft of the manuscript; all authors contributed to the final draft of the manuscript.

## Pre-publication history

The pre-publication history for this paper can be accessed here:



## Supplementary Material

Additional File 1 Statement by MRCP(UK) concerning recruitment, training and monitoring of examiners Click here for file
