# Supplementary material for: Assessment of examiner leniency and stringency ('hawk-dove effect') in the MRCP(UK) clinical examination (PACES) using multi-facet Rasch modelling
Source: BMC Med Educ. 2006 Aug 18;6:42. doi: 10.1186/1472-6920-6-42 (PMC1569374; doi:10.1186/1472-6920-6-42)
Supplement: Additional File 1 — Statement by MRCP(UK) concerning recruitment, training and monitoring of examiners [file 1472-6920-6-42-S1.pdf]

MRCP(UK) Part 2 Clinical Examination (PACES) Examiners

Recruitment

Appointments are made by open advertisement and by nominations from existing Examiners. MRCP(UK) Clinical Examiners are appointed after consideration of an application form and testimonials from two examiners.

The criteria for appointment of examiners to MRCP(UK) Clinical Examiners status:

- To be involved in and have demonstrated an interest in medical education;
- To hold the Fellowship of the Royal College of Physicians of London or the Royal College of Physicians of Edinburgh or the Royal College of Physicians and Surgeons of Glasgow (consultants or specialist registrars
- To be practising in some aspect of general medicine with additional subspecialty or area of interest.
- To observe a Clinical Examination and go through a course of training. A briefing is conducted before each Examination for all Examiners and a programme for training in the regions has commenced;
- To make themselves available at least once a year (for three days) to carry out clinical examining duties;
- To maintain a satisfactory standard.

We also monitor whether examiners have undergone equal opportunity training

Induction / Training

Examiners who have been selected by the nominations panel are provided with information about their role, the Examination and two training videos. Examiners are required to attend an examination centre for one day, and on the basis of their performance and knowledge of the Examination the NVE decides whether they suitable.

At the Examination a trainee Examiner undertakes all the processes carried out by examiners, except that the marksheets completed by the trainee does not affect the candidates' scores. This involves one cycle (five candidates) at a Talking Station, and one cycle (five candidates) at a Clinical Station. In centres running three cycles per day, the trainee also spends one cycle either as the 11th Examiner or observing one candidate at each Station to gain an overview of the whole exam.

Records

The Colleges maintain a record on each Examiner, showing the date of their induction, dates of examining and hosting, dates they were a Nominated Visiting Examiner, dates and nature of re-training, dates and nature of any untoward incidents. The Colleges will also be responsible for keeping an up-to-date record of examiners' eligibility, so that ineligible examiners do not examine.

Policy on poorly performing Examiners

Examiners who are identified by the MRCP(UK) Clinical Examining Board as having performed poorly are given a period of instruction monitored by a member of the Clinical Examining Board. This may involve them having to repeat the process of training, observation and induction that they go through on appointment. Failure to respond appropriately to the remedial action taken will lead to their removal from the list of Examiners.

An examiner can also be summarily terminated for a gross infringement.
